# Impact of academic collaboration and quality of clinical orthopaedic research conducted in low- and middle-income countries

**DOI:** 10.1051/sicotj/2016042

**Published:** 2017-01-30

**Authors:** Hao-Hua Wu, Max Liu, Kushal R. Patel, Wes Turner, Lincoln Baltus, Amber M. Caldwell, Jesse C. Hahn, Ralph Richard Coughlin, Saam Morshed, Theodore Miclau, David W. Shearer

**Affiliations:** 1 Department of Orthopaedic Surgery, Orthopaedic Trauma Institute, Institute for Global Orthopaedics and Traumatology, University of California, San Francisco San Francisco CA 94110 USA; 2 Department of Orthopaedic Surgery, University of Illinois College of Medicine Chicago IL 60612 USA

**Keywords:** Orthopaedics, Global surgery, Low- and middle-income Countries, Clinical research, Academic collaboration, Levels of evidence, Research capacity

## Abstract

*Background*: Little is known about the quality of orthopaedic investigations conducted in low- and middle-income countries (LMICs). Academic collaboration is one model to build research capacity and improve research quality. Our study aimed to determine (1) the quality of clinical orthopaedic research conducted in LMICs, (2) the World Bank Regions and LMICs that publish the highest quality studies, (3) the pattern of collaboration among investigators and (4) whether academic collaboration between LMIC and non-LMIC investigators is associated with studies that have higher levels of evidence.

*Methods*: Orthopaedic studies from 2004 to 2014 conducted in LMICs were extracted from multiple electronic databases. The World Bank Region, level of evidence and author country-affiliation were recorded. Collaboration was defined as a study that included an LMIC with non-LMIC investigator.

*Results*: There were 958 studies that met inclusion criteria of 22,714 searched. Ninety-seven (10.1%) of included studies achieved Level 1 or 2 evidence, but case series (52.3%) were the most common. Collaboration occurred in 14.4% of studies and the vast majority of these (88.4%) were among academic institutions. Collaborative studies were more likely to be Level 1 or 2 (20.3% vs. 8.4%, *p* < 0.01), prospective (34.8% vs. 22.9% *p* = 0.04) and controlled (29.7% vs. 14.4%, *p* < 0.01) compared to non-collaborative studies.

*Conclusions*: Although orthopaedic studies in LMICs rarely reach Level 1 or 2 evidence, studies published through academic collaboration between LMIC and non-LMIC investigators are associated with higher levels of evidence and more prospective, controlled designs.

## Introduction

There is increasing recognition of the importance of musculoskeletal disease as a global health issue, particularly as it relates to injury. An estimated 1.2 million deaths and 50 million nonfatal injuries occur each year due to road traffic accidents alone [1], with traumatic injuries contributing to more global disability than human immunodeficiency virus (HIV), tuberculosis and malaria combined [2, 3]. The burden of injury is greatest in low- and middle-income countries (LMICs), where resource-limited orthopaedic surgeons face a daunting volume of musculoskeletal disease [2, 4]. Efforts to address this disparity in care have manifested through surgical missions, donated implants and educational programmes [4, 5]. Less, however, is known about the impact of clinical research [4, 5]. Recent literature suggests that clinical orthopaedic research conducted in LMICs may help to answer relevant clinical questions and shape public health policy [4]. Although a growing body of research exists to inform treatment for orthopaedic disease in high-income countries (HICs), generalizing results to LMICs is difficult due to delays in treatment, differences in training and availability of equipment [6, 7]. Thus, it would be ideal for orthopaedic surgeons practicing in LMICs to base their treatment on high-quality research conducted in a similar setting [4].

To date, the quality of clinical research conducted in LMICs has not been assessed. Orthopaedic surgeons weigh a study’s level of evidence (LOE) before determining how the results apply clinical practice [8–10], as Level 1 or 2 studies can potentially reveal important therapeutic, diagnostic, prognostic and economic outcomes [11, 12]. Given the importance of study quality in shaping treatment protocol, the state of orthopaedic research in developing countries requires comprehensive review. Some have hypothesized that collaboration between investigators from LMICs and non-LMICs (e.g. upper-middle and high-income countries) can improve study quality [4, 13]. While there is growing evidence that investigators from LMICs strive to answer clinically relevant questions through research, they may lack resources, training and protected time away from clinical responsibilities [14]. However, collaboration with academically affiliated non-LMIC authors may help local investigators overcome these barriers through funding support, research education and division of labour [4]. Thus, investigations are needed to evaluate how international academic partnership can improve study quality.

Our study aimed to determine (1) the quality of clinical orthopaedic research conducted in LMICs, (2) the World Bank Regions and LMICs that publish the highest quality studies, (3) the pattern of collaboration among investigators and (4) whether academic collaboration between LMIC and non-LMIC investigators is associated with studies that have higher levels of evidence.

## Material and methods

We conducted a scoping review using the Arksey and O’Malley framework with modifications from Levac et al. and Daudt et al. [15–17]. Five authors sought and assessed orthopaedic studies conducted in LMICs that were published between June 2004 and June 2014. The LMICs were defined as any country listed as a “Low-income” or “Lower-middle-income” economy according to the 2014 World Bank classification [18]. Eighty-two countries met our study’s definition of LMIC (Table 1). The studies were identified by comprehensive textword and MeSH-based electronic searches of PubMed/MEDLINE, EMBASE and Cochrane library that was developed with assistance from a research librarian. Our search strategy combined terms for orthopaedic surgery, LMICs, musculoskeletal injury, musculoskeletal anatomy and human studies to be as inclusive as possible (Appendix).

**Table 1.** Countries with low-income or lower-middle-income economies as defined by The World Bank. Table adapted from http://data.worldbank.org/about/country-and-lending-groups.

Low-income economies ($1045 or less) [*n* = 31]		
Afghanistan	Gambia, The	Niger
Benin	Guinea	Rwanda
Burkina Faso	Guinea-Bisau	Sierra Leone
Burundi	Haiti	Somalia
Cambodia	Korea, Dem Rep.	South Sudan
Central African Republic	Liberia	Tanzania
Chad	Madagascar	Togo (Sub-Saharan)
Comoros	Malawi	Uganda
Congo, Dem. Rep	Mali	Zimbabwe
Eritrea	Mozambique	
Ethiopia	Nepal	
Lower-middle-income economies ($1046 to $4125) [*n* = 51]		
Armenia	Indonesia	Samoa
Bangladesh	Kenya	São Tomé and Principe
Bhutan	Kiribati	Senegal
Bolivia	Kosovo	Solomon Islands
Cabo Verde	Kyrgyz Republic	Sri Lanka
Cameroon	Lao PDR	Sudan
Congo, Rep.	Lesotho	Swaziland
Côte d’Ivoire	Mauritania	Syrian Arab Republic
Djibouti	Micronesia, Fed. Sts.	Tajikistan
Egypt, Arab Rep.	Mongolia	Timor-Leste
El Salvador	Morocco (North Africa)	Ukraine
Georgia	Myanmar	Uzbekistan (Central Asia)
Ghana	Nicaragua	Vanuatu
Guatemala	Nigeria (West Africa)	Vietnam
Guyana	Pakistan (South Asia)	West Bank and Gaza
Honduras	Papua New Guinea	Yemen, Rep.
India	Philippines	Zambia

Full-text articles were assessed for eligibility in the order of title, abstract and manuscript. Included studies (1) deal primarily with a low- or lower-middle-income country (LMIC), (2) pertain to orthopaedic surgery, (3) enrol humans and (4) were original peer-reviewed publications. Studies that reported three cases or fewer, were non-English and pertained to a high-income country (HIC) at war, animals, biomechanics or laboratory values were excluded.

Each included study was read in its entirety and a REDCap survey for data extraction was created that identified the study location, author’s academic affiliation and study quality. Study location and author affiliations were categorized into eight World Bank Regions: East Asia and Pacific, Europe and Central Asia, Latin America and the Caribbean, Middle East and North Africa, Latin America and the Caribbean, Middle East and North Africa, North America, South Asia (Table 2). Collaboration was determined by the investigator country-affiliation and defined as LMIC-only, Multicentre (LMIC with LMIC investigator), Collaborative (LMIC with non-LMIC investigator) and non-LMIC (e.g. upper-middle or high-income country investigators) only. Academic partnerships were defined as a study where the authors from both LMICs and non-LMICs were affiliated with an academic institution.

**Table 2.** Countries by World Bank Regions, adapted from http://data.worldbank.org/about/country-and-lending-groups.

East Asia and Pacific, *n* = 37		
American Samoa	Korea, Rep.	Philippines
Australia	Lao PDR	Samoa
Brunei Darussalam	Macao SAR, China	Singapore
Cambodia	Malaysia	Solomon Islands
China	Marshall Islands	Taiwan, China
Fiji	Micronesia, Fed. Sts.	Thailand
French Polynesia	Mongolia	Timor-Leste
Guam	Myanmar	Tonga
Hong Kong SAR, China	New Caledonia	Tuvalu
Indonesia	New Zealand	Vanuatu
Japan	Northern Mariana Islands	Vietnam
Kiribati	Palau	
Korea, Dem. Rep.	Papua New Guinea	
Europe and Central Asia, *n* = 57		
Albania	Germany	Netherlands
Andorra	Greece	Norway
Armenia	Greenland	Poland
Austria	Hungary	Portugal
Azerbaijan	Iceland	Romania
Belarus	Ireland	Russian Federation
Belgium	Isle of Man	San Marino
Bosnia and Herzegovina	Italy	Serbia
Bulgaria	Kazakhstan	Slovak Republic
Channel Islands	Kosovo	Slovenia
Croatia	Kyrgyz Republic	Spain
Cyprus	Latvia	Sweden
Czech Republic	Liechtenstein	Switzerland
Denmark	Lithuania	Tajikistan
Estonia	Luxembourg	Turkey
Faeroe Islands	Macedonia, FYR	Turkmenistan
Finland	Moldova	Ukraine
France	Monaco	United Kingdom
Georgia	Montenegro	Uzbekistan
Latin America and the Caribbean, *n* = 41		
Antigua and Barbuda	Dominica	Peru
Argentina	Dominican Republic	Puerto Rico
Aruba	Ecuador	Sint Maarten (Dutch part)
Bahamas, The	El Salvador	St. Kitts and Nevis
Barbados	Grenada	St. Lucia
Belize	Guatemala	St. Martin (French part)
Bolivia	Guyana	St. Vincent and the Grenadines
Brazil	Haiti	Suriname
Cayman Islands	Honduras	Trinidad and Tobago
Chile	Jamaica	Turks and Caicos Islands
Colombia	Mexico	Uruguay
Costa Rica	Nicaragua	Venezuela, RB
Cuba	Panama	Virgin Islands (U.S.)
Curacao	Paraguay	
Middle East and North Africa, *n* = 21		
Algeria	Jordan	Qatar
Bahrain	Kuwait	Saudi Arabia
Djibouti	Lebanon	Syrian Arab Republic
Egypt, Arab Rep.	Libya	Tunisia
Iran, Islamic Rep.	Malta	United Arab Emirates
Iraq	Morocco	West Bank and Gaza
Israel	Oman	Yemen, Rep.
North America, *n* = 3		
Bermuda	Canada	United States
South Asia, *n* = 8		
Afghanistan	India	Pakistan
Bangladesh	Maldives	Sri Lanka
Bhutan	Nepal	
Sub-Saharan Africa, *n* = 48		
Angola	Gabon	Nigeria
Benin	Gambia, The	Rwanda
Botswana	Ghana	São Tomé and Principe
Burkina Faso	Guinea	Senegal
Burundi	Guinea-Bissau	Seychelles
Cabo Verde	Kenya	Sierra Leone
Cameroon	Lesotho	Somalia
Central African Republic	Liberia	South Africa
Chad	Madagascar	South Sudan
Comoros	Malawi	Sudan
Congo, Dem. Rep.	Mali	Swaziland
Congo, Rep	Mauritania	Tanzania
Côte d’Ivoire	Mauritius	Togo
Equatorial Guinea	Mozambique	Uganda
Eritrea	Namibia	Zambia
Ethiopia	Niger	Zimbabwe

Finally, the study quality was assessed by the levels of evidence (LOE), presence of control group, prospective or retrospective design, type of study and presence of randomization. Due to the heterogeneity of study design in the global orthopaedic literature, which includes qualitative and epidemiologic studies that cannot be categorized with the LOE scale, no other quality assessment tools were utilized. To determine the level of evidence of each study, we used the 2015 scale adopted by the Journal of Bone and Joint Surgery and derived from recommendations given by the Centre for Evidence-Based Medicine in Oxford, United Kingdom [19, 20]. The LOE scale divides studies into four categories: Diagnostic, Prognostic, Therapeutic, and Economic and Decision Analyses. Each category can then be subdivided into Level 1–5 evidence, with each level having its own definition [19]. Level 1 and 2 studies were considered high levels of evidence, while those of Level 3, 4 and 5 were considered low levels of evidence. Studies that had no levels of evidence, such as epidemiologic or qualitative studies, were also noted. LOE was used as the primary indicator of study quality due to its ubiquitous use as a validated measure of study strength [9, 11, 21–23]. In addition, studies that do include control groups, collect data prospectively and randomize interventions have been shown to produce higher quality evidence than studies that do not [8, 10].

### Data analysis

Descriptive statistics were used to summarize all data. Discrete variables were summarized as counts or proportions, and skewed continuous variables were reported as medians with interquartile ranges (IQRs). Chi-square analysis was used to determine the association between nominal variables. Significance was set at *p* < 0.05.

## Results

**Figure 1.**
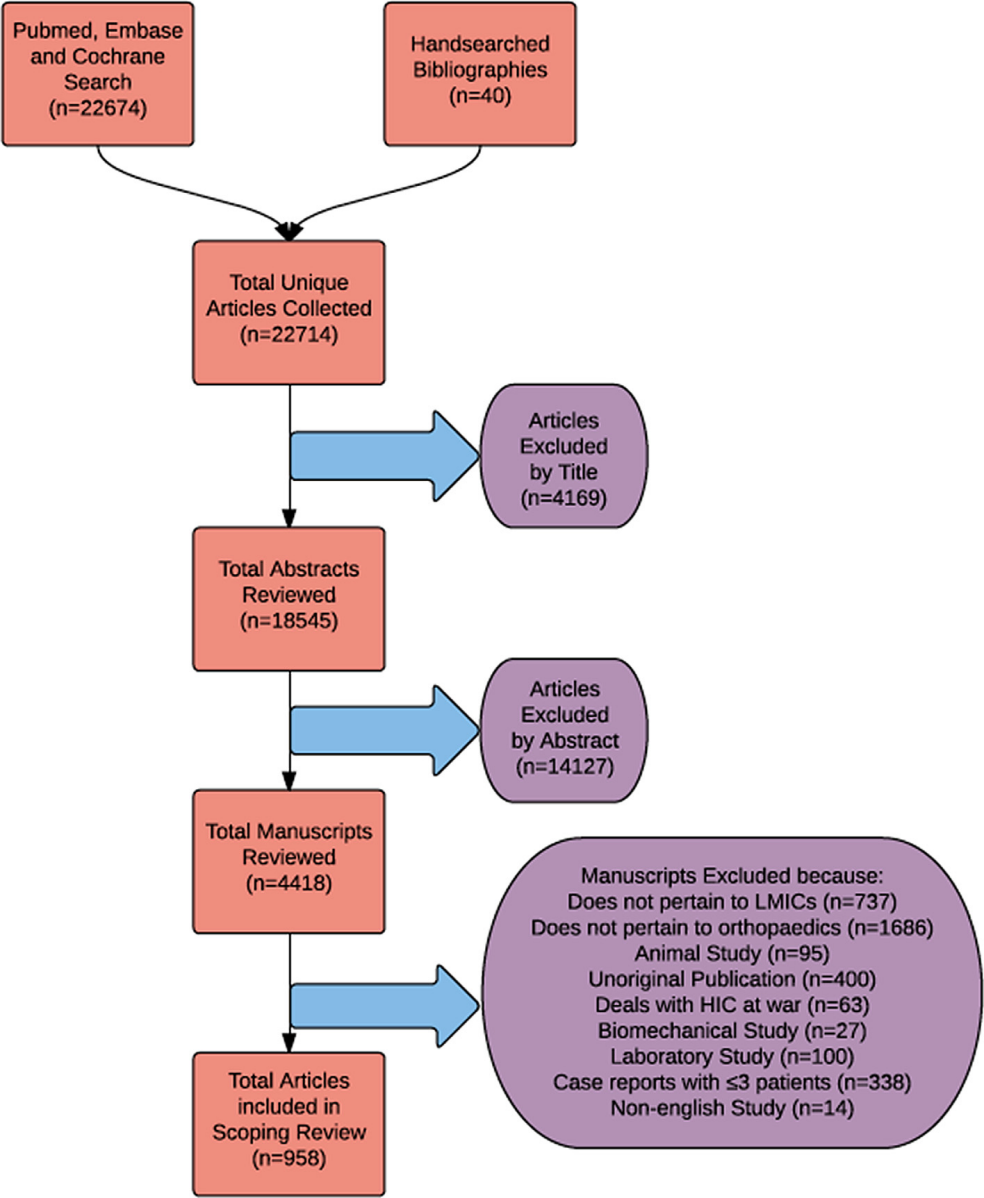
Flowchart shows included and excluded studies from search to data extraction.

**Figure 2.**
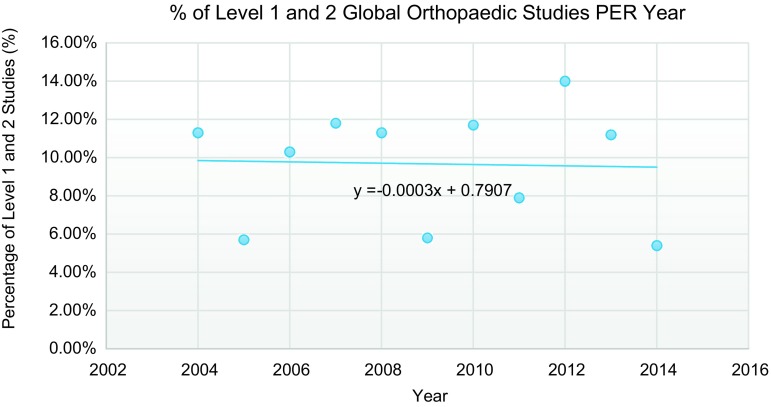
Percentage of Level 1 and 2 global orthopaedic studies conducted in low- and middle-income countries per year from June 2004 to June 2014.

**Figure 3.**
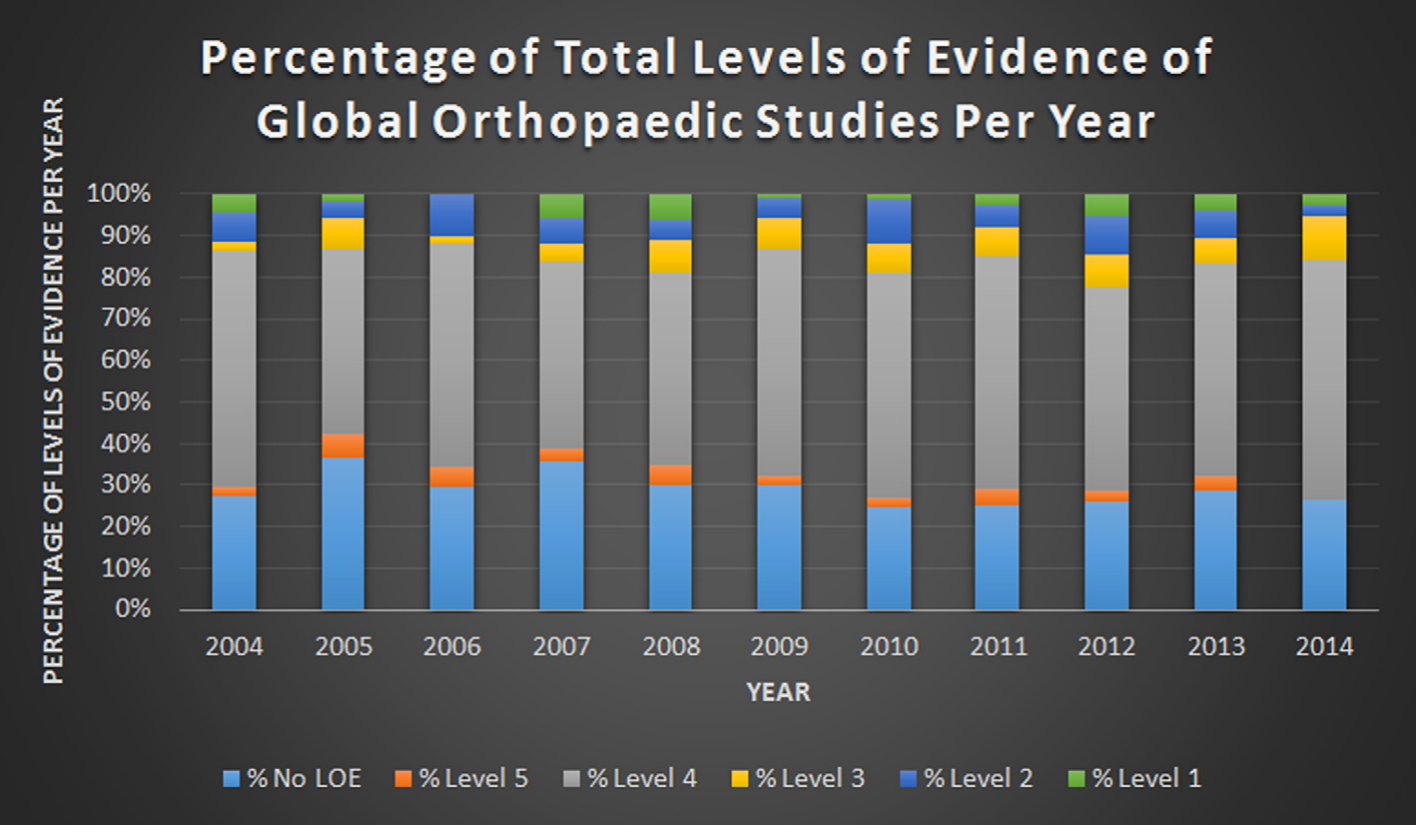
Percentage of total levels of evidence of global orthopaedic studies conducted in low- and middle-income countries per year from June 2004 June 2014.

Out of 22,714 unique articles assessed, 958 met all inclusion criteria (Figure 1). Over the past decade, 265 (27.6%) of clinical orthopaedic research studies conducted in LMICs were epidemiologic or qualitative and had no LOE. Of the clinical studies that could be assessed with levels of evidence, only 97 (10.1%) were Level 1 or 2. Studies were most commonly designed as prospective or retrospective case series (501 [52.3%]). In addition, only 158 (16.5%) of all studies had a control group and only 24 (2.5%) underwent randomization. Overall, the majority of data from these studies (75.4%) were collected retrospectively. From 2004 to 2014, the median percentage of global orthopaedic studies published per year with a high LOE (e.g. Level 1 and 2 studies) was 11.2% (IQR: 5.8–11.7). The percentage of high-quality global orthopaedics being conducted in LMICs annually has been stagnant over time (Figure 2), even though the number of LMIC orthopaedic studies has steadily increased over the same time period. A chi-square contingency table shows no statistically significant difference in the percentage of Level 1 and 2 studies published over the past decade (*p* = 0.4) (Figure 3). Of note, out of 503 therapeutic studies, 374 studies recommended the use of tested treatment or intervention, even though most of the studies 394 (78.3%) were Levels 4 and 5 with respect to evidence.

**Figure 4.**
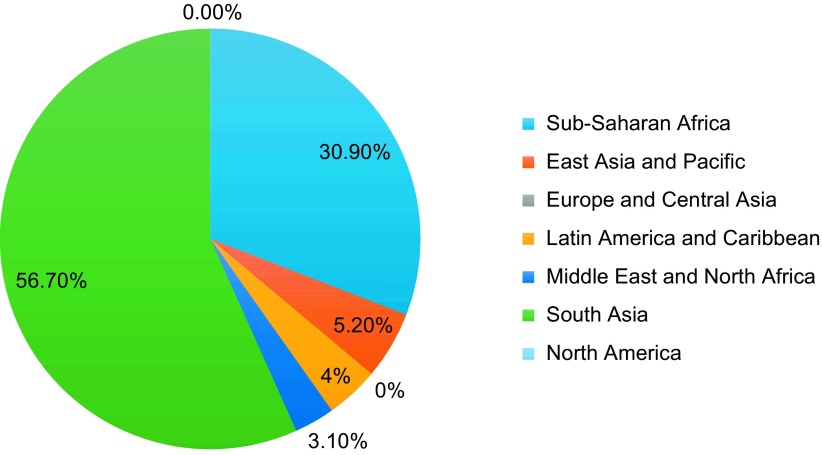
Percentage of high-quality studies (e.g. Levels 1 and 2) published by World Bank Region from 2004 to 2014.

Most studies took place in South Asia (48.4%) and Sub-Saharan Africa (32.6%). Of the 97 studies that were Level 1 or 2 evidence, 55 (56.7%) took place in South Asia and 30 (30.9%) took place in Sub-Saharan Africa (Figure 4). Pakistan (25), India (19), Nigeria (14) and Nepal (9) were the LMICs with the most Level 1 and 2 studies published in the past decade (Table 3). Out of the 82 countries classified as LMICs by the World Bank, only 23 (28.0%) had published a Level 1 or 2 orthopaedic study in the past decade. LMICs from the Middle East and North Africa (3.1%), Latin America and Caribbean (4%) and East Asia and Pacific (5.2%) produced the lowest percentage of Level 1 and 2 studies since 2004.

**Table 3.** Study setting region, Level 1 and 2 studies by region and countries that have published Level 1 and 2 studies.

Variables	Study characteristics (*n* = 958)
Study setting region	
Sub-Saharan Africa (*n* [%])	312 [32.6%]
Level 1 or 2 studies (*n* [%])	30 [30.9%]
Number of Level 1 or 2 orthopaedic studies published by country since 2004	Nigeria (14), Malawi (5), Sudan (3), Cameroon (2), Kenya (2) Ethiopia (1), Sierra Leone (1), Tanzania (1), Uganda (1)
East Asia and Pacific (*n* [%])	53 [5.5%]
Level 1 or 2 studies (*n* [%])	5 [5.2%]
Number of Level 1 or 2 orthopaedic studies published by country since 2004	Cambodia (1), Mongolia (1), Myanmar (1), Philippines (1), Vietnam (1)
Europe and Central Asia (*n* [%])	17 [1.8%]
Level 1 or 2 studies (*n* [%])	0 [0%]
Latin America and Caribbean (*n* [%])	30 [3.1%]
Level 1 or 2 studies (*n* [%])	4 [4.1%]
Number of Level 1 or 2 orthopaedic studies published by country since 2004	Haiti (2), Guatemala (1), Guyana (1)
Middle East and North Africa (*n* [%])	82 [8.6%]
Level 1 or 2 studies (*n* [%])	3 [3.1]
Number of Level 1 or 2 orthopaedic studies published by country since 2004	Egypt (3)
South Asia (*n* [%])	464 [48.4%]
Level 1 or 2 studies (*n* [%])	55 [56.7%]
Number of Level 1 or 2 orthopaedic studies published by country since 2004	Pakistan (25), India (19), Nepal (9), Bangladesh (2), Afghanistan (1)

**Figure 5.**
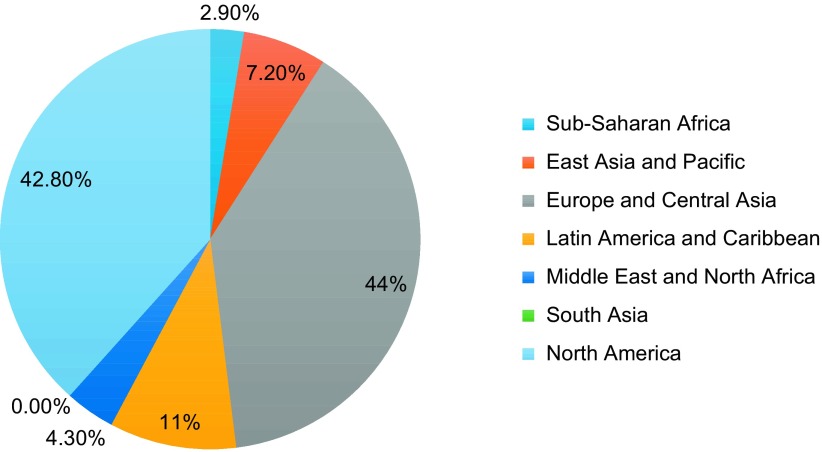
Percentage of non-LMIC collaborative authors by World Bank Region from 2004 to 2014.

Over the past decade, 138 (14.4%) of global orthopaedic studies have been Collaborative, 30 (3.1%) have been Multicentre, 85 (8.9%) have only included authors from non-LMICs and 705 (73.6%) have only included authors from LMICs. The majority (75.8%) of them took place in academically affiliated institutions. In addition, the majority of non-LMIC authors practised either in Europe and Central Asia (44%) or in North America (42.8%) (Figure 5).

Out of the 138 studies where LMIC and non-LMIC authors worked together, 122 (88.4%) of them represented academic partnerships. Collaborative studies were more likely to be Level 1 or 2 (20.3% vs. 8.4%, *p* < 0.01), prospective (34.8% vs. 22.9% *p* = 0.04) and controlled (29.7% vs. 14.4%, *p* < 0.01) compared to non-collaborative studies (Table 4). There was no difference in randomization between Collaborative and non-collaborative studies (*p* = 0.53).

**Table 4.** Characteristics of collaborative global orthopaedic studies from 2004 to 2014.

Variables	Study characteristics (*n* = 138)
Region of collaborating site by WHO classification	
Sub-Saharan Africa (*n* [%])	4 [2.9%]
North America (*n* [%])	59 [42.8%]
East Asia and Pacific (*n* [%])	10 [7.2%]
Europe and Central Asia (*n* [%])	60 [43.5%]
Middle East and North Africa (*n* [%])	6 [4.3%]
Latin America and Caribbean (*n* [%])	15 [10.9%]
South Asia (*n* [%])	0 [0%]
Quality of collaborative studies	
Level 1 and 2 studies (*n* [%])	28 (20.3%)
Level 3, 4, 5 studies (*n* [%])	57 (41.3%)
No Level of Evidence (*n* [%])	53 (38.4%)
Controls (*n* [%])	41 [29.7%]
Prospective (*n* [%])	48 (34.8%)
Randomized (*n* [%])	1 (0.7%)

## Discussion

While the importance of evidence-based clinical practice is increasingly recognized in high-income countries, there is an enormous gap in the volume and quality of musculoskeletal research relevant to clinicians and patients in LMICs. Our study shows that the majority of clinical orthopaedic researches published in LMICs are designed as case series, and these studies rarely attain high levels of evidence, a phenomenon that has persisted over the past decade. Notably, there appears to be no significant difference between the proportion of Level 1 and 2 studies published in 2004 and the years leading up to 2014. Studies with low levels of evidence cannot accurately determine the treatment outcome and may be harmful if misinterpreted [11]. Our data shows that while the majority of therapeutic studies conducted in LMICs recommended the use of the tested intervention, they were mostly designed as case series. Given the importance of research in the effort to treat global orthopaedic disease, efforts to enable local researchers to produce high-quality studies are warranted.

One factor associated with higher quality research is academic collaboration. Our data suggest that while studies produced by collaboration between LMIC and non-LMIC investigators account for less than 15% of total research, they are associated with higher levels of evidence and more prospective, controlled designs.

Our finding that orthopaedic research conducted in LMICs was unlikely to attain high levels of evidence is similar to the literature published in major Western journals. For instance, Reich et al. evaluated seven years’ worth of publications from *The Journal of Bone and Joint Surgery* (American) and found that only one of the Level 1 and 2 studies published was conducted in an LMIC [12]. In addition, the proportion of high level studies published in LMICs does not compare favorably with that of non-LMIC studies. An analysis of three American journals found that 23% of studies published in the last decade achieved Level 1 or 2 evidence [9], more than double the proportion of Level 1 and 2 LMIC-conducted studies during the same time frame. Notably, our data also suggests that the overall quality of LMIC publications has not improved over the past decade, which lies in stark contrast to studies of high impact journals that suggest the number of high-quality studies has increased significantly over time [9, 12]. Future research into why a significant gap in research quality exists between high-resource and low-resource environments is warranted.

Even though the quality of research from LMICs is lower overall, studies conducted in South Asia and Sub-Saharan Africa were more likely to attain high levels of evidence. Notably, Pakistan, India, Nigeria and Nepal produced over two-thirds of Level 1 and 2 studies in the past decade. In contrast, 59 of the world’s 82 LMICs did not produce a single Level 1 or 2 study over the same time frame. This finding is similar to recent literature that indirectly suggests LMICs from South Asia produce the highest quality studies in Western journals compared to other LMIC regions [12]. However, it is still unknown why this disparity by region exists, which warrants further investigation.

Collaboration between LMIC and non-LMIC investigators is associated with significantly higher quality of published clinical research with respect to levels of evidence, prospective design and presence of control groups. Notably, over 88% of these studies represented academic partnerships, in which both the LMIC and non-LMIC investigators were affiliated with an academic institution. Although, it is unclear why Collaborative studies are associated with higher research quality, recent literature has hypothesized that investigators from high-income countries can enable their counterparts in LMICs to conduct research through longitudinal partnership [4, 13, 24]. For example, Morshed et al. outline a potential model of partnership in which investigators from an academic HIC institution helped LMIC-based surgeons develop a prospective cohort study through assistance in protocol development, research training, funding and resource procurement [4]. Academic affiliation of LMIC partners may also be an important ingredient for successful partnerships, since academic advancement and research requirement for trainees are often cited by LMIC authors as incentives for research participation [4, 14].

Our study had several limitations. First, given the heterogeneity of included articles, we were unable to apply additional standardized quality assessment tools, which may have affected our ability to gauge the study quality. However, LOE has been shown to be a validated measure of study quality and have adequate interobserver reliability [9, 21]. Second, our review only included studies since June 2004 and could have been strengthened by including more publication years. However, our study provides a snapshot of study quality over the last decade and is by far the most comprehensive existing review of the global orthopaedics literature. Third, as a literature review, our study design cannot prove causal inference exists between academic collaboration and study quality. Our data does, however, show that non-LMIC and LMIC collaboration is associated with higher study quality, which warrants further investigation. Finally, our study did not include primarily non-English publications, which may have excluded high-quality studies published in other languages. However, recent literature suggests that language restrictions of reviews may not produce meaningful bias [25], although future studies with broader language requirements are needed.

## Conclusion

Recent literature has shown that many investigators who practice in LMICs have a strong interest in participating in research studies [14]. However, surgeons in LMICs contend with a lack of formalized research training, resources and protected time as significant barriers to improving study quality [4, 14]. These barriers may explain why clinical orthopaedic research conducted in LMICs rarely attains high levels of evidence compared to those conducted in non-LMICs, a phenomenon that has not improved in the past decade. Thus, more research is needed to determine how LMIC investigators can best overcome impediments to high-quality research in resource-poor environments. Academic collaboration is one of the most promising solutions, as it is associated with higher quality studies published over the last decade. Thus, greater investment of resources into developing and investigating the impact of academic collaboration is warranted.

## Funding

No funding to disclose.

## Conflict of interest

Dr. Coughlin reports AAOS-International Committee Institute for Global Orthopaedics and Traumatology/UCSF: Board or committee member.

Dr. Morshed reports personal fees from Microbion, grants from Philips outside the submitted work; Orthopaedic Research Society: Board or committee member; Orthopaedic Trauma Association: Board or committee member.

Dr. Miclau reports personal fees from Acelity, personal fees from Amgen Co, grants from Baxter, grants from Synthes outside the submitted work; Inman Abbott Society: Board or committee member; Orthopaedic Research Society: Board or committee member; Orthopaedic Trauma Association: Board or committee member; Osteosynthesis and Trauma Care Foundation: Board or committee member.

The other co-authors of this study declare no conflict of interest for this project.

## Appendix

### Search strategy

1. orthopedics[mh] OR orthopedic procedures[mh] OR fractures, bone[mh] OR musculoskeletal diseases[mh:noexp] OR bone diseases[mh] OR cartilage diseases[mh:noexp] OR chondromalacia patellae[mh] OR osteochondritis[mh] OR polychondritis, relapsing[mh] OR foot deformities[mh] OR foot diseases[mh] OR hand deformities[mh] OR joint diseases[mh] OR muscular diseases[mh:noexp] OR arthrogryposis[mh] OR compartment syndromes[mh] OR contracture[mh] OR medial tibial stress syndrome[mh] OR musculoskeletal pain[mh] OR tendinopathy[mh] OR musculoskeletal abnormalities[mh:noexp] OR campomelic dysplasia[mh] OR hip dislocation, congenital[mh] OR klippel-feil syndrome[mh] OR limb deformities, congenital[mh] OR synostosis[mh] OR rheumatic diseases[mh:noexp] OR osteoarthritis[mh] OR tennis elbow[mh] OR amputation, traumatic[mh] OR arm injuries[mh] OR back injuries[mh] OR craniocerebral trauma[mh:noexp] OR dislocations[mh] OR fractures, cartilage[mh] OR hand injuries[mh] OR hip injuries[mh] OR leg injuries[mh] OR ligaments, articular[majr] OR limb salvage[mh] OR muscle, skeletal[majr] OR musculoskeletal system[mh:noexp] OR orthopedic equipment[mh] OR skeleton/injuries[mh] OR skeleton/surgery[mh] OR spinal injuries[mh] OR spine/injuries[mh] OR tendon injuries[mh] OR tendons[majr]
2. amput*[tiab] OR dislocation*[tiab] OR fracture[tiab] OR fractures[tiab] OR musculoskeletal*[tiab] OR orthoped*[tiab] OR orthopaed*[tiab]
3. accidents[mh] OR accident*[tiab] OR injur*[tiab] OR polytrauma*[tiab] OR “wounds and injuries”[majr:noexp] OR wounds, gunshot[mh] OR blast injuries[mh]
4. bone[tiab] OR bones[tiab] OR humerus[tiab] OR humeri[tiab] OR ulna[tiab] OR ulnas[tiab] OR ulnae[tiab] OR scaphoid[tiab] OR scaphoids[tiab] OR vertebra*[tiab] OR spine[tiab] OR spines[tiab] OR pelvis*[tiab] OR pelves[tiab] OR femur[tiab] OR femurs[tiab] OR tibia[tiab] OR tibias[tiab] OR fibula[tiab] OR fibulas[tiab] OR talus[tiab] OR tali[tiab] OR calcaneus[tiab] OR calcanei[tiab] OR calcanea[tiab] OR shoulder[tiab] OR shoulders[tiab] OR elbow[tiab] OR elbows[tiab] OR wrist[tiab] OR wrists[tiab] OR hip[tiab] OR hips[tiab] OR knee[tiab] OR knees[tiab] OR ankle[tiab] OR ankles[tiab] OR extremity[tiab] OR extremities[tiab] OR “open injury”[tiab] OR “open injuries”[tiab]
5. 3 AND 4
6. 1 OR 2 OR 5
7. Afghanistan[Mesh] OR Bangladesh[Mesh] OR Benin[Mesh] OR Burkina Faso[Mesh] OR Burundi[Mesh] OR Cambodia[Mesh] OR Central African Republic[Mesh] OR Chad[Mesh] OR Comoros[Mesh] OR Democratic Republic of the Congo[Mesh] OR Eritrea[Mesh] OR Ethiopia[Mesh] OR Gambia[Mesh] OR Guinea[Mesh] OR Guinea-Bissau[Mesh] OR Haiti[Mesh] OR Kenya[Mesh] OR Democratic People’s Republic of Korea[Mesh] OR Kyrgyzstan[Mesh] OR Liberia[Mesh] OR Madagascar[Mesh] OR Malawi[Mesh] OR Mali[mh] OR Mozambique[Mesh] OR Myanmar[Mesh] OR Nepal[Mesh] OR Niger[Mesh] OR Rwanda[Mesh] OR Sierra Leone[Mesh] OR Somalia[Mesh] OR Sudan[Mesh] OR Tajikistan[Mesh] OR Tanzania[Mesh] OR Togo[Mesh] OR Uganda[Mesh] OR Zimbabwe[Mesh]
8. Armenia[Mesh] OR Bhutan[Mesh] OR Bolivia[Mesh] OR Cameroon[Mesh] OR Cape Verde[Mesh] OR Congo[Mesh] OR Cote d’Ivoire[Mesh] OR Djibouti[Mesh] OR Egypt[Mesh] OR El Salvador[Mesh] OR “Georgia (Republic)”[Mesh] OR Ghana[Mesh] OR Guatemala[Mesh] OR Guyana[Mesh] OR Honduras[Mesh] OR Indonesia[Mesh] OR India[Mesh] OR Kosovo[Mesh] OR Laos[Mesh] OR Lesotho[Mesh] OR Mauritania[Mesh] OR Moldova[Mesh] OR Mongolia[Mesh] OR Morocco[Mesh] OR Nicaragua[Mesh] OR Nigeria[Mesh] OR Pakistan[Mesh] OR Papua New Guinea[Mesh] OR Paraguay[Mesh] OR Philippines[Mesh] OR Samoa[Mesh:noexp] OR Independent State of Samoa[mh] OR Senegal[Mesh] OR Sri Lanka[Mesh] OR Sudan[Mesh] OR Swaziland[Mesh] OR Syria[Mesh] OR East Timor[Mesh] OR Ukraine[Mesh] OR Uzbekistan[Mesh] OR Vanuatu[Mesh] OR (Vietnam[Mesh] NOT veteran*) OR Yemen[Mesh] OR Zambia[Mesh]
9. Afghani*[tiab] OR Bangladesh*[tiab] OR Benin[tiab] OR “Burkina Faso”[tiab] OR Burundi*[tiab] OR Cambodia*[tiab] OR “Central African Republic”[tiab] OR Chad[tiab] OR Comoros[tiab] OR (Congo[tiab] NOT “congo red”) OR Congolese[tiab] OR Zaire[tiab] OR Eritrea*[tiab] OR Ethiopia*[tiab] OR Gambia*[tiab] OR (Guinea*[tiab] NOT (guinea fowl* OR guinea pig* OR “new guinea”)) OR “Guinea-Bissau”[tiab] OR Haiti*[tiab] OR Kenya*[tiab] OR “Democratic People’s Republic of Korea”[tiab] OR “North Korea”[tiab] OR North Korean*[tiab] OR Kyrgyz*[tiab] OR Liberia*[tiab] OR Madagascar[tiab] OR Malawi[tiab] OR Malawian*[tiab] OR Mali[tiab] OR Mozambique[tiab] OR Myanmar[tiab] OR Burma[tiab] OR Burmese[tiab] OR Nepal[tiab] OR Nepalese[tiab] OR Niger[tiab] OR Rwanda*[tiab] OR “Sierra Leone”[tiab] OR Somalia[tiab] OR Somali[tiab] OR Somalis[tiab] OR “South Sudan”[tiab] OR Tajikistan*[tiab] OR Tadjikistan*[tiab] OR Tanzania*[tiab] OR Zanzibar*[tiab] OR Tanganyika[tiab] OR Togo[tiab] OR Togolese[tiab] OR Uganda*[tiab] OR Zimbabw*[tiab] OR (Rhodesia[tiab] NOT “Rhodesian ridgeback”)
10. Armenia*[tiab] OR Bhutan*[tiab] OR Bolivia*[tiab] OR Cameroon*[tiab] OR “Cape Verde”[tiab] OR “Cote d’Ivoire”[tiab] OR “Ivory Coast”[tiab] OR Djibouti*[tiab] OR Egypt*[tiab] OR El Salvador*[tiab] OR “Georgia Republic”[tiab] OR “Republic of Georgia”[tiab] OR Ghana*[tiab] OR Guatemal*[tiab] OR Guyana[tiab] OR Guyanese[tiab] OR “British Guiana”[tiab] OR Hondur*[tiab] OR (India[tiab] NOT (“india ink” OR “indian ink”)) OR “Asian Indian”[tiab] OR “Asian Indians”[tiab] OR Indonesia*[tiab] OR Kiribati[tiab] OR Kosovo[tiab] OR Kosovan*[tiab] OR Kosovar*[tiab] OR Laos[tiab] OR “Lao PDR” OR “LAO People’s Democratic Republic”[tiab] OR Laotian*[tiab] OR Lesotho*[tiab] OR Mauritania*[tiab] OR (Micronesia*[tiab] AND “Federated States”[tiab]) OR Moldova*[tiab] OR Mongolia*[tiab] OR Morocc*[tiab] OR Nicaragua*[tiab] OR Nigeria*[tiab] OR Pakistan*[tiab] OR “Papua New Guinea”[tiab] OR Paraguay[tiab] OR Paraguayan*[tiab] OR Philippines[tiab] OR (Filipino*[tiab] OR Filipina*[tiab] NOT (Filipino American* OR United States[mh])) OR (Samoa*[tiab] NOT American Samoa*) OR “Sao Tome”[tiab] OR Senegal[tiab] OR Senegalese[tiab] OR Solomon Island*[tiab] OR “Sri Lanka”[tiab] OR Sri Lankan*[tiab] OR Ceylon[tiab] OR Sudan*[tiab] OR Swaziland[tiab] OR Syria*[tiab] OR “Timor-Leste”[tiab] OR “East Timor”[tiab] OR Ukrain*[tiab] OR Uzbekistan*[tiab] OR Vanuatu*[tiab] OR (Vietnam*[tiab] NOT veteran*) OR “West Bank”[tiab] OR Gaza[tiab] OR Palestin*[tiab] OR Yemen*[tiab] OR Zambia*[tiab]
11. developing countries[mh] OR “developing country”[tiab] OR “developing countries”[tiab] OR LMIC[tiab] OR LMICs[tiab] OR “low income countries”[tiab] OR “low income country”[tiab] OR “low and middle income countries”[tiab] OR “low and middle income country”[tiab] OR “lower middle income countries”[tiab] OR “lower middle income country”[tiab] OR “developing nation”[tiab] OR “developing nations”[tiab] OR “developing world”[tiab] OR “developing economy”[tiab] OR “developing economies”[tiab] OR “transitional country”[tiab] OR “transitional countries”[tiab] OR “global burden”[tiab] OR “global health”[tiab] OR global orthop*[tiab] OR “global outreach”[tiab] OR “global public health”[tiab] OR (global[ti] AND watch[ti]) OR “international health”[tiab] OR “international public health”[tiab] OR world health[majr] OR international cooperation[majr] OR “resource poor”[tiab] OR austere environment*[tiab] OR “third world”[tiab]
12. Africa[mh:noexp] OR Africa, Central[mh:noexp] OR Africa, Eastern[mh:noexp] OR Africa, Northern[mh:noexp] OR Africa South of the Sahara[mh:noexp] OR Africa, Southern[mh:noexp] OR Africa, Western[mh:noexp] OR central Africa*[ti] OR east Africa*[ti] OR eastern Africa*[ti] OR north Africa*[ti] OR northern Africa*[ti] OR southern Africa*[ti] OR west africa*[ti] OR western africa*[ti] OR sahara*[ti] OR subsahara*[ti] OR (Asia[mh:noexp] NOT (china[mh] OR japan[mh] OR Singapore[mh] OR south korea[mh])) OR Asia, Central[mh:noexp] OR Asia, Southeastern[mh:noexp] OR Asia, Western[mh:noexp] OR central asia*[ti] OR south asia*[ti] OR south asia*[ti] OR southeast asia*[ti] OR southeastern asia*[ti] OR southern asia*[ti] OR west asia*[tiab] OR western asia*[ti] OR Central America[mh:noexp] OR central America*[ti] OR Europe, Eastern[mh:noexp] OR eastern Europe*[ti] OR South America[mh:noexp] OR South America*[ti] OR Caribbean Region[mh:noexp] OR Caribbean[ti] OR Middle East[mh:noexp]
13. 7 OR 8 OR 9 OR 10 OR 11 OR 12
14. 7 OR 8 OR 9 OR 10 OR 11 OR 12 NOT (animals[mh] NOT humans[mh]) NOT Dental journals[sb] NOT News[pt] NOT (mummies OR mummy OR history, ancient[mh] OR paleoanthro* OR paleoepidem* OR paleopath* OR paleont* OR archeolog* OR ancient egypt* OR dynast* OR fossil* OR forensic anthropology[mh] OR history of medicine[mh])
15. 14 AND (“2004”[Date - Publication]: “3000”[Date - Publication])
16. 14 AND (“2004”[Date - Publication]: “3000”[Date - Publication]) AND English[Language]

### Pubmed and Cochrane Search (June 1, 2014)

(orthopedics[mh] OR orthopedic procedures[mh] OR fractures, bone[mh] OR musculoskeletal diseases[mh:noexp] OR bone diseases[mh] OR cartilage diseases[mh:noexp] OR chondromalacia patellae[mh] OR osteochondritis[mh] OR polychondritis, relapsing[mh] OR foot deformities[mh] OR foot diseases[mh] OR hand deformities[mh] OR joint diseases[mh] OR muscular diseases[mh:noexp] OR arthrogryposis[mh] OR compartment syndromes[mh] OR contracture[mh] OR medial tibial stress syndrome[mh] OR musculoskeletal pain[mh] OR tendinopathy[mh] OR musculoskeletal abnormalities[mh:noexp] OR campomelic dysplasia[mh] OR hip dislocation, congenital[mh] OR klippel-feil syndrome[mh] OR limb deformities, congenital[mh] OR synostosis[mh] OR rheumatic diseases[mh:noexp] OR osteoarthritis[mh] OR tennis elbow[mh] OR amputation, traumatic[mh] OR arm injuries[mh] OR back injuries[mh] OR craniocerebral trauma[mh:noexp] OR dislocations[mh] OR fractures, cartilage[mh] OR hand injuries[mh] OR hip injuries[mh] OR leg injuries[mh] OR ligaments, articular[majr] OR limb salvage[mh] OR muscle, skeletal[majr] OR musculoskeletal system[mh:noexp] OR orthopedic equipment[mh] OR skeleton/injuries[mh] OR skeleton/surgery[mh] OR spinal injuries[mh] OR spine/injuries[mh] OR tendon injuries[mh] OR tendons[majr] OR amput*[tiab] OR dislocation*[tiab] OR fracture[tiab] OR fractures[tiab] OR musculoskeletal*[tiab] OR orthoped*[tiab] OR orthopaed*[tiab] OR ((accidents[mh] OR accident*[tiab] OR injur*[tiab] OR polytrauma*[tiab] OR “wounds and injuries”[majr:noexp] OR wounds, gunshot[mh] OR blast injuries[mh]) AND (bone[tiab] OR bones[tiab] OR humerus[tiab] OR humeri[tiab] OR ulna[tiab] OR ulnas[tiab] OR ulnae[tiab] OR scaphoid[tiab] OR scaphoids[tiab] OR vertebra*[tiab] OR spine[tiab] OR spines[tiab] OR pelvis*[tiab] OR pelves[tiab] OR femur[tiab] OR femurs[tiab] OR tibia[tiab] OR tibias[tiab] OR fibula[tiab] OR fibulas[tiab] OR talus[tiab] OR tali[tiab] OR calcaneus[tiab] OR calcanei[tiab] OR calcanea[tiab] OR shoulder[tiab] OR shoulders[tiab] OR elbow[tiab] OR elbows[tiab] OR wrist[tiab] OR wrists[tiab] OR hip[tiab] OR hips[tiab] OR knee[tiab] OR knees[tiab] OR ankle[tiab] OR ankles[tiab] OR extremity[tiab] OR extremities[tiab] OR “open injury”[tiab] OR “open injuries”[tiab]))) AND ((Afghanistan[Mesh] OR Bangladesh[Mesh] OR Benin[Mesh] OR Burkina Faso[Mesh] OR Burundi[Mesh] OR Cambodia[Mesh] OR Central African Republic[Mesh] OR Chad[Mesh] OR Comoros[Mesh] OR Democratic Republic of the Congo[Mesh] OR Eritrea[Mesh] OR Ethiopia[Mesh] OR Gambia[Mesh] OR Guinea[Mesh] OR Guinea-Bissau[Mesh] OR Haiti[Mesh] OR Kenya[Mesh] OR Democratic People’s Republic of Korea[Mesh] OR Kyrgyzstan[Mesh] OR Liberia[Mesh] OR Madagascar[Mesh] OR Malawi[Mesh] OR Mali[mh] OR Mozambique[Mesh] OR Myanmar[Mesh] OR Nepal[Mesh] OR Niger[Mesh] OR Rwanda[Mesh] OR Sierra Leone[Mesh] OR Somalia[Mesh] OR Sudan[Mesh] OR Tajikistan[Mesh] OR Tanzania[Mesh] OR Togo[Mesh] OR Uganda[Mesh] OR Zimbabwe[Mesh] OR Armenia[Mesh] OR Bhutan[Mesh] OR Bolivia[Mesh] OR Cameroon[Mesh] OR Cape Verde[Mesh] OR Congo[Mesh] OR Cote d’Ivoire[Mesh] OR Djibouti[Mesh] OR Egypt[Mesh] OR El Salvador[Mesh] OR “Georgia (Republic)”[Mesh] OR Ghana[Mesh] OR Guatemala[Mesh] OR Guyana[Mesh] OR Honduras[Mesh] OR Indonesia[Mesh] OR India[Mesh] OR Kosovo[Mesh] OR Laos[Mesh] OR Lesotho[Mesh] OR Mauritania[Mesh] OR Moldova[Mesh] OR Mongolia[Mesh] OR Morocco[Mesh] OR Nicaragua[Mesh] OR Nigeria[Mesh] OR Pakistan[Mesh] OR Papua New Guinea[Mesh] OR Paraguay[Mesh] OR Philippines[Mesh] OR Samoa[Mesh:noexp] OR Independent State of Samoa[mh] OR Senegal[Mesh] OR Sri Lanka[Mesh] OR Sudan[Mesh] OR Swaziland[Mesh] OR Syria[Mesh] OR East Timor[Mesh] OR Ukraine[Mesh] OR Uzbekistan[Mesh] OR Vanuatu[Mesh] OR (Vietnam[Mesh] NOT veteran*) OR Yemen[Mesh] OR Zambia[Mesh] OR Afghani*[tiab] OR Bangladesh*[tiab] OR Benin[tiab] OR “Burkina Faso”[tiab] OR Burundi*[tiab] OR Cambodia*[tiab] OR “Central African Republic”[tiab] OR Chad[tiab] OR Comoros[tiab] OR (Congo[tiab] NOT “congo red”) OR Congolese[tiab] OR Zaire[tiab] OR Eritrea*[tiab] OR Ethiopia*[tiab] OR Gambia*[tiab] OR (Guinea*[tiab] NOT (guinea fowl* OR guinea pig* OR “new guinea”)) OR “Guinea-Bissau”[tiab] OR Haiti*[tiab] OR Kenya*[tiab] OR “Democratic People’s Republic of Korea”[tiab] OR “North Korea”[tiab] OR North Korean*[tiab] OR Kyrgyz*[tiab] OR Liberia*[tiab] OR Madagascar[tiab] OR Malawi[tiab] OR Malawian*[tiab] OR Mali[tiab] OR Mozambique[tiab] OR Myanmar[tiab] OR Burma[tiab] OR Burmese[tiab] OR Nepal[tiab] OR Nepalese[tiab] OR Niger[tiab] OR Rwanda*[tiab] OR “Sierra Leone”[tiab] OR Somalia[tiab] OR Somali[tiab] OR Somalis[tiab] OR “South Sudan”[tiab] OR Tajikistan*[tiab] OR Tadjikistan*[tiab] OR Tanzania*[tiab] OR Zanzibar*[tiab] OR Tanganyika[tiab] OR Togo[tiab] OR Togolese[tiab] OR Uganda*[tiab] OR Zimbabw*[tiab] OR (Rhodesia[tiab] NOT “Rhodesian ridgeback”) OR Armenia*[tiab] OR Bhutan*[tiab] OR Bolivia*[tiab] OR Cameroon*[tiab] OR “Cape Verde”[tiab] OR “Cote d’Ivoire”[tiab] OR “Ivory Coast”[tiab] OR Djibouti*[tiab] OR Egypt*[tiab] OR El Salvador*[tiab] OR “Georgia Republic”[tiab] OR “Republic of Georgia”[tiab] OR Ghana*[tiab] OR Guatemal*[tiab] OR Guyana[tiab] OR Guyanese[tiab] OR “British Guiana”[tiab] OR Hondur*[tiab] OR (India[tiab] NOT (“india ink” OR “indian ink”)) OR “Asian Indian”[tiab] OR “Asian Indians”[tiab] OR Indonesia*[tiab] OR Kiribati[tiab] OR Kosovo[tiab] OR Kosovan*[tiab] OR Kosovar*[tiab] OR Laos[tiab] OR “Lao PDR” OR “LAO People’s Democratic Republic”[tiab] OR Laotian*[tiab] OR Lesotho*[tiab] OR Mauritania*[tiab] OR (Micronesia*[tiab] AND “Federated States”[tiab]) OR Moldova*[tiab] OR Mongolia*[tiab] OR Morocc*[tiab] OR Nicaragua*[tiab] OR Nigeria*[tiab] OR Pakistan*[tiab] OR “Papua New Guinea”[tiab] OR Paraguay[tiab] OR Paraguayan*[tiab] OR Philippines[tiab] OR (Filipino*[tiab] OR Filipina*[tiab] NOT (Filipino American* OR United States[mh])) OR (Samoa*[tiab] NOT American Samoa*) OR “Sao Tome”[tiab] OR Senegal[tiab] OR Senegalese[tiab] OR Solomon Island*[tiab] OR “Sri Lanka”[tiab] OR Sri Lankan*[tiab] OR Ceylon[tiab] OR Sudan*[tiab] OR Swaziland[tiab] OR Syria*[tiab] OR “Timor-Leste”[tiab] OR “East Timor”[tiab] OR Ukrain*[tiab] OR Uzbekistan*[tiab] OR Vanuatu*[tiab] OR (Vietnam*[tiab] NOT veteran*) OR “West Bank”[tiab] OR Gaza[tiab] OR Palestin*[tiab] OR Yemen*[tiab] OR Zambia*[tiab]) OR (developing countries[mh] OR “developing country”[tiab] OR “developing countries”[tiab] OR LMIC[tiab] OR LMICs[tiab] OR “low income countries”[tiab] OR “low income country”[tiab] OR “low and middle income countries”[tiab] OR “low and middle income country”[tiab] OR “lower middle income countries”[tiab] OR “lower middle income country”[tiab] OR “developing nation”[tiab] OR “developing nations”[tiab] OR “developing world”[tiab] OR “developing economy”[tiab] OR “developing economies”[tiab] OR “transitional country”[tiab] OR “transitional countries”[tiab] OR “global burden”[tiab] OR “global health”[tiab] OR global orthop*[tiab] OR “global outreach”[tiab] OR “global public health”[tiab] OR (global[ti] AND watch[ti]) OR “international health”[tiab] OR “international public health”[tiab] OR world health[majr] OR international cooperation[majr] OR “resource poor”[tiab] OR austere environment*[tiab] OR “third world”[tiab]) OR (Africa[mh:noexp] OR Africa, Central[mh:noexp] OR Africa, Eastern[mh:noexp] OR Africa, Northern[mh:noexp] OR Africa South of the Sahara[mh:noexp] OR Africa, Southern[mh:noexp] OR Africa, Western[mh:noexp] OR central Africa*[ti] OR east Africa*[ti] OR eastern Africa*[ti] OR north Africa*[ti] OR northern Africa*[ti] OR southern Africa*[ti] OR west africa*[ti] OR western africa*[ti] OR sahara*[ti] OR subsahara*[ti] OR (Asia[mh:noexp] NOT (china[mh] OR japan[mh] OR Singapore[mh] OR south korea[mh])) OR Asia, Central[mh:noexp] OR Asia, Southeastern[mh:noexp] OR Asia, Western[mh:noexp] OR central asia*[ti] OR south asia*[ti] OR south asia*[ti] OR southeast asia*[ti] OR southeastern asia*[ti] OR southern asia*[ti] OR west asia*[tiab] OR western asia*[ti] OR Central America[mh:noexp] OR central America*[ti] OR Europe, Eastern[mh:noexp] OR eastern Europe*[ti] OR South America[mh:noexp] OR South America*[ti] OR Caribbean Region[mh:noexp] OR Caribbean[ti] OR Middle East[mh:noexp])) NOT (animals[mh] NOT humans[mh]) NOT Dental journals[sb] NOT News[pt] NOT (mummies OR mummy OR history, ancient[mh] OR paleoanthro* OR paleoepidem* OR paleopath* OR paleont* OR archeolog* OR ancient egypt* OR dynast* OR fossil* OR forensic anthropology[mh] OR history of medicine[mh]) AND (“2004”[Date - Publication]: “3000”[Date - Publication]) AND English[Language].

### Embase Search (June 1, 2014)

‘orthopedics’/de OR ‘orthopedic surgery’/exp OR ‘fracture’/exp OR ‘musculoskeletal disease’/de OR ‘arthropathy’/exp OR ‘bone disease’/exp OR ‘chondropathy’/exp OR ‘compartment syndrome’/exp OR ‘contracture’/mj OR ‘flexion contracture’/de OR ‘hip contracture’/de OR ‘joint contracture’/de OR ‘muscle contracture’/de OR ‘tendon contracture’/de OR ‘dupuytren contracture’/de OR ‘enthesopathy’/exp OR ‘limb disease’/de OR ‘arm disease’/exp OR ‘leg disease’/exp OR ‘limb defect’/de OR ‘limb deformity’/de OR ‘limb injury’/exp OR ‘limb malformation’/exp OR ‘limb pain’/de OR ‘limb tumor’/de OR ‘muscle disease’/mj OR ‘musculoskeletal injury’/exp OR ‘musculoskeletal pain’/exp OR ‘musculoskeletal system malformation’/exp/mj OR ‘rheumatic disease’/de OR ‘tendinitis’/exp OR ‘ligament’/exp/mj OR ‘musculoskeletal system’/mj OR ‘orthopedic equipment’/exp/mj OR ‘pelvis injury’/exp OR ‘skeletal muscle’/exp/mj OR ‘tendon’/exp/mj OR amput*:ab,ti OR back NEAR/3 injur* OR dislocation*:ab,ti OR fracture:ab,ti OR fractures:ab,ti OR musculoskelet*:ab,ti OR orthopedi*:ab,ti OR orthopaedi*:ab,ti OR (‘accident’/exp OR ‘accidental injury’/de OR accident*:ab,ti OR injur*:ab,ti OR polytrauma*:ab,ti OR ‘injury’/mj OR ‘blunt trauma’/exp OR ‘crush trauma’/de OR ‘multiple trauma’/de AND (bone:ab,ti OR bones:ab,ti OR humerus:ab,ti OR humeri:ab,ti OR ulna:ab,ti OR ulnas:ab,ti OR ulnae:ab,ti OR scaphoid:ab,ti OR scaphoids:ab,ti OR vertebra*:ab,ti OR spine:ab,ti OR spines:ab,ti OR pelvis*:ab,ti OR pelves:ab,ti OR femur:ab,ti OR femurs:ab,ti OR tibia:ab,ti OR tibias:ab,ti OR fibula:ab,ti OR fibulas:ab,ti OR talus:ab,ti OR tali:ab,ti OR calcaneus:ab,ti OR calcanei:ab,ti OR calcanea:ab,ti OR shoulder:ab,ti OR shoulders:ab,ti OR elbow:it,ab OR elbows:ab,ti OR wrist:ab,ti OR wrists:ab,ti OR hip:ab,ti OR hips:ab,ti OR knee:ab,ti OR knees:ab,ti OR ankle:ab,ti OR ankles:ab,ti OR extremity:ab,ti OR extremities:ab,ti OR ‘open injury’:ab,ti OR ‘open injuries’:ab,ti)) AND (‘afghanistan’/de OR ‘bangladesh’/de OR ‘benin’/de OR ‘burkina faso’/de OR ‘burundi’/de OR ‘cambodia’/de OR ‘central african republic’/de OR ‘chad’/de OR ‘comoros’/de OR ‘democratic republic congo’/de OR ‘eritrea’/de OR ‘ethiopia’/de OR ‘gambia’/de OR ‘guinea’/de OR ‘guinea-bissau’/de OR ‘haiti’/de OR ‘kenya’/de OR ‘north korea’/de OR ‘kyrgyzstan’/de OR ‘liberia’/de OR ‘madagascar’/de OR ‘malawi’/de OR ‘mali’/de OR ‘mozambique’/de OR ‘myanmar’/de OR ‘nepal’/de OR ‘niger’/de OR ‘rwanda’/de OR ‘sierra leone’/de OR ‘somalia’/de OR ‘tajikistan’/de OR ‘tanzania’/de OR ‘togo’/de OR ‘uganda’/de OR ‘zimbabwe’/de OR ‘armenia’/de OR ‘bhutan’/de OR ‘bolivia’/de OR ‘cameroon’/de OR ‘cape verde’/de OR ‘congo’/de OR ‘cote d`ivoire’/exp OR ‘djibouti’/de OR ‘egypt’/de OR ‘el salvador’/de OR ‘georgia (republic)’/de OR ‘ghana’/de OR ‘guatemala’/de OR ‘guyana’/de OR ‘honduras’/de OR ‘indonesia’/de OR ‘india’/de OR ‘kosovo’/de OR ‘laos’/de OR ‘lesotho’/de OR ‘mauritania’/de OR ‘federated states of micronesia’/de OR ‘moldova’/de OR ‘mongolia’/de OR ‘morocco’/de OR ‘nicaragua’/de OR ‘nigeria’/de OR ‘pakistan’/de OR ‘papua new guinea’/de OR ‘paraguay’/de OR ‘philippines’/de OR ‘samoa’/de OR ‘sao tome and principe’/de OR ‘senegal’/de OR ‘solomon islands’/de OR ‘sri lanka’/de OR ‘sudan’/de OR ‘swaziland’/de OR ‘syrian arab republic’/de OR ‘timor-leste’/de OR ‘ukraine’/de OR ‘uzbekistan’/de OR ‘vanuatu’/de OR (‘vietnam’/de NOT veteran*) OR ‘palestine’/de OR ‘yemen’/de OR ‘zambia’/de OR afghani*:ab,ti OR bangladesh*:ab,ti OR benin:ab,ti OR ‘burkina faso’:ab,ti OR burundi*:ab,ti OR cambodia*:ab,ti OR ‘central african republic’:ab,ti OR chad:ab,ti OR comoros:ab,ti OR (congo:ab,ti NOT ‘congo red’) OR congolese:ab,ti OR zaire:ab,ti OR eritrea*:ab,ti OR ethiopia*:ab,ti OR gambia*:ab,ti OR (guinea*:ab,ti NOT (guinea NEXT/1 fowl* OR guinea NEXT/1 pig* OR ‘guinea pig’/exp OR ‘new guinea’)) OR ‘guinea-bissau’:ab,ti OR haiti*:ab,ti OR kenya*:ab,ti OR ‘north korea’:ab,ti OR ‘north korean’:ab,ti OR ‘north koreans’:ab,ti OR ‘kyrgyz republic’:ab,ti OR kyrgyzstan*:ab,ti OR liberia*:ab,ti OR madagascar:ab,ti OR malawi:ab,ti OR mali:ab,ti OR mozambique:ab,ti OR myanmar:ab,ti OR burma:ab,ti OR burmese:ab,ti OR nepal:ab,ti OR nepalese:ab,ti OR niger:ab,ti OR rwanda*:ab,ti OR ‘sierra leone’:ab,ti OR somalia:ab,ti OR somali:ab,ti OR somalis:ab,ti OR ‘south sudan’:ab,ti OR tajikistan*:ab,ti OR tadjikistan*:ab,ti OR tanzania*:ab,ti OR zanzibar*:ab,ti OR tanganyika:ab,ti OR togo:ab,ti OR togolese:ab,ti OR uganda*:ab,ti OR zimbabw*:ab,ti OR (rhodesia:ab,ti NOT ‘rhodesian ridgeback’) OR armenia*:ab,ti OR bhutan*:ab,ti OR bolivia*:ab,ti OR cameroon*:ab,ti OR ‘cape verde’:ab,ti OR ‘cote d ivoire’:ab,ti OR ‘ivory coast’:ab,ti OR djibouti*:ab,ti OR egypt*:ab,ti OR ‘el salvador’:ab,ti OR ‘el salvadoran’:ab,ti OR ‘el salvadorans’:ab,ti OR ‘georgia republic’:ab,ti OR ‘republic of georgia’:ab,ti OR ghana*:ab,ti OR guatemal*:ab,ti OR guyana:ab,ti OR guyanese:ab,ti OR ‘british guiana’:ab,ti OR hondur*:ab,ti OR (india:ab,ti NOT (‘india ink’ OR ‘indian ink’)) OR ‘asian indian’:ab,ti OR ‘asian indians’:ab,ti OR indonesia*:ab,ti OR kiribati:ab,ti OR kosovo:ab,ti OR laos:ab,ti OR ‘lao pdr’ OR laotian*:ab,ti OR lesotho*:ab,ti OR mauritania*:ab,ti OR (micronesia:ab,ti AND ‘federated states’) OR moldova:ab,ti OR mongolia*:ab,ti OR morocc*:ab,ti OR nicaragua*:ab,ti OR nigeria*:ab,ti OR pakistan*:ab,ti OR ‘papua new guinea’:ab,ti OR paraguay:ab,ti OR philippines:ab,ti OR (filipino*:ab,ti OR filipina*:ab,ti NOT (‘filipino american’ OR ‘filipino americans’ OR ‘filipina american’ OR ‘filipina americans’ OR ‘united states’/exp)) OR (samoa:ab,ti OR samoan*:ab,ti NOT american NEXT/1 samoa*) OR ‘sao tome’:ab,ti OR senegal:ab,ti OR senegalese:ab,ti OR ‘solomon island’:ab,ti OR ‘solomon islands’:ab,ti OR ‘sri lanka’:ab,ti OR ‘sri lankan’:ab,ti OR ‘sri lankans’:ab,ti OR ceylon:ab,ti OR sudan*:ab,ti OR swaziland:ab,ti OR syria*:ab,ti OR ‘timor-leste’:ab,ti OR ‘east timor’:ab,ti OR ukrain*:ab,ti OR uzbekistan*:ab,ti OR vanuatu*:ab,ti OR (vietnam*:ab,ti NOT veteran*) OR ‘west bank’:ab,ti OR gaza:ab,ti OR palestin*:ab,ti OR yemen*:ab,ti OR zambia*:ab,ti OR ‘developing country’/de OR ‘developing country’:ab,ti OR ‘developing countries’:ab,ti OR lmic:ab,ti OR lmics:ab,ti OR ‘low income countries’:ab,ti OR ‘low income country’:ab,ti OR ‘low and middle income countries’:ab,ti OR ‘low and middle income country’:ab,ti OR ‘lower middle income countries’:ab,ti OR ‘lower middle income country’:ab,ti OR ‘developing nation’:ab,ti OR ‘developing nations’:ab,ti OR ‘developing world’:ab,ti OR ‘developing economy’:ab,ti OR ‘developing economies’:ab,ti OR ‘transitional country’:ab,ti OR ‘transitional countries’:ab,ti OR ‘global burden’:ab,ti OR ‘global health’:ab,ti OR (global NEXT/1 orthop*):ab,ti OR ‘global outreach’:ab,ti OR ‘global public health’:ab,ti OR ‘global watch’:ab,ti OR ‘international health’:ab,ti OR ‘international public health’:ab,ti OR ‘international cooperation’/mj OR ‘resource poor’:ab,ti OR ‘austere environment’:ab,ti OR ‘third world’:ab,ti OR ‘africa’/de OR ‘central africa’/de OR ‘north africa’/de OR ‘africa south of the sahara’/de OR (central NEXT/1 africa*):ti OR (east NEXT/1 africa*):ti OR (eastern NEXT/1 africa*):ti OR (north NEXT/1 africa*):ti OR (northern NEXT/1 africa*):ti OR (southern NEXT/1 africa*):ti OR sahara*:ti OR subsahara*:ti OR (west NEXT/1 africa*):ti OR (western NEXT/1 africa*):ti OR (‘asia’/mj NOT (‘china’/de OR ‘japan’/de OR ‘singapore’/de OR ‘south korea’/de)) OR ‘south asia’/de OR ‘southeastern asia’ OR (central NEXT/1 asia*):ti OR (south NEXT/1 asia*):ti OR (southeast* NEXT/1 asia*):ti OR (southern NEXT/1 asia*):ti OR (west NEXT/1 asia*):ti OR (western NEXT/1 asia*):ti OR ‘central america’/de OR (central NEXT/1 america*):ti OR ‘eastern europe’/de OR (eastern NEXT/1 europe*):ti OR ‘south america’/de OR ‘south and central america’/de OR (south NEXT/1 america*):ti OR ‘caribbean islands’/de OR caribbean:ti OR ‘middle east’/de) NOT ([animals]/lim NOT ([humans]/lim OR ‘patient’/exp)) NOT (‘mummies’/exp OR ‘mummy’/exp OR ‘ancient history’ OR paleo* OR archeolog* OR ancient NEXT/1 egypt* OR dynast* OR ‘forensic anthropology’/exp OR ‘history of medicine’/exp OR ‘history’/exp) AND [english]/lim AND [2004-2014]/py.

### Cochrane Library Search (June 1, 2014)

orthop* or fracture* or musculoskeletal or amput* or dislocat* or extremity or extremities or bone or bones or ankle or calcaneus or elbow or femur or fibula or femur or foot or hand or hip or humerus or knee or leg or pelvis or scaphoid or shoulder or spine or talus or tibia or ulna or vertebrae or wrist or open next injur*:ti,ab,kw and Afghani* or Bangladesh* or Benin or “Burkina Faso” or Burundi* or Cambodia* or “Central African Republic” or Chad or Comoros or (Congo* not “congo red”) or Zaire or Eritrea* or Ethiopia* or Gambia* or (Guinea not (guinea next fowl* or guinea next pig* or “new guinea”)) or “Guinea-Bissau” or Haiti* or Kenya* or (Democratic next People* and “Republic of Korea”) or North next Korea* or Kyrgyz* or Liberia or Madagascar or Malawi* or Mali or Mozambique or Myanmar or Burma or Burmese or Nepal or Nepalese or Niger or Rwanda* or “Sierra Leone” or Somali* or “South Sudan” or Tajikistan* or Tadjikistan* or Tanzania* or Zanzibar* or Tanganyika or Togo or Togolese or Uganda* or Zimbabw* or (Rhodesia* not “Rhodesian ridgeback”) or Armenia* or Bhutan* or Bolivia* or Cameroon* or “Cape Verde” or “Cote d Ivoire” or “Ivory Coast” or Djibouti* or El next Salvador* or “Georgia Republic” or “Republic of Georgia” or Ghana* or Guatemala* or Guyana or Guyanese or Hondur* or Indonesia* or Kiribati or Kosovo* or Kosovar* or Laos or “Lao PDR” or LAO next People* or Laotian* or Lesotho* or Mauritania* or “Federated States of Micronesia” or Moldova* or Mongolia* or Morocco* or Nicaragua* or Nigeria* or Pakistan* or “Papua New Guinea” or Paraguay* or Philippines or (Filipino* or Filipina* not (Filipino next American* or “United States”)) or (Samoa* not “American Samoa”) or “Sao Tome” or Senegal* or Solomon next Island* or Sri next Lanka* or Ceylon or Sudan* or Swaziland or Syria* or “Timor-Leste” or “East Timor” or Ukrain* or Uzbekistan* or Vanuatu* or (Vietnam* not veteran*) or “West Bank” or “Gaza” or Palestin* or Yemen* or Zambia* or developing next countr* or LMIC or LMICs or “low income countries” or “low income country” or “low and middle income countries” or “low and middle income country” or “lower middle income countries” or “lower middle income country” or developing next nation* or developing next world or “developing economy” or “developing economies” or transitional next countr* or “global burden” or “global health” or global next orthop* or “global outreach” or “global public health” or global next watch or “international health” or “international public health” “world health” or “resource poor” or austere environment* or “third world” or central next Africa* or east next Africa* or eastern next Africa* or north next Africa* or northern next Africa* or southern next Africa* or west next africa* or western next africa* or sahara* or subsahara* or central next asia* or south next asia* or south next asia* or southeast next asia* or southeastern next asia* or southern next asia* or west next asia* or western next asia* or central next America* or eastern next Europe* or South next America* Publication Date from 2004 to 2014 (Word variations have been searched).

## References

[1] World Health Organization (2015) Global status report on road safety time for action. Available at: http://whqlibdoc.who.int/publications/2009/9789241563840_eng.pdf. Accessed November 10, 2015.

[2] Gosselin RA, Spiegel DA, Coughlin R, Zirkle LG (2009) Injuries: the neglected burden in developing countries. Bulletin of the WHO 87, 246. 10.2471/BLT.08.052290 PMC2672580 19551225

[3] Krug E (2012) Decade of action for road safety 2011–2020. Injury 43, 6–7. 22153116 10.1016/j.injury.2011.11.002

[4] Morshed S, Shearer DW, Coughlin RR. 2013 (Collaborative partnerships and the future of global orthopaedics) Clin Orthop Relat Res 471, 3088–3092. 23884799 10.1007/s11999-013-3145-x PMC3773130

[5] Chen AT, Pedtke A, Kobs JK, Edwards GS Jr, Coughlin RR, Gosselin RA (2012) Volunteer orthopedic surgical trips in Nicaragua: a cost-effectiveness evaluation. World J Surg 36(12), 2802–2808. 22777413 10.1007/s00268-012-1702-1

[6] Dormans JP, Fisher RC, Pill SG (2001) Orthopaedics in the developing world: present and future concerns. J Am Acad Orthop Surg 9(5), 289–296. 11575908 10.5435/00124635-200109000-00002

[7] Museru LM, McHaro CN. 2002 (The dilemma of fracture treatment in developing countries) Int Orthop 26(6), 324–327. 12466862 10.1007/s00264-002-0408-7 PMC3620973

[8] Bernstein J (2004) Evidence-based medicine. J Am Acad Orthop Surg 12, 80–88. 15089081 10.5435/00124635-200403000-00003

[9] Grant HS, Tjoumakaris FP, Maltenfort MG, Freedman KB (2014) Levels of evidence in the clinical sports medicine literature: are we getting better over time? Am J Sports Med 42(7), 1738–1742. 24758781 10.1177/0363546514530863

[10] Sackett DL, Rosenberg WM, Gray JA, Haynes RB, Richardson WS (1996) Evidence based medicine: what it is and what it isn’t. BMJ 312(7023), 71–72. 8555924 10.1136/bmj.312.7023.71 PMC2349778

[11] Burns PB, Rohrich RJ, Chung KC (2012) The levels of evidence and their role in evidence-based medicine. Plast Reconstr Surg 128(1), 305–310. 10.1097/PRS.0b013e318219c171 PMC3124652 21701348

[12] Reich MS, Shaw J, Barrett I, Goldberg VM, Schnaser E (2014) Level of evidence trends in the Journal of Bone and Joint Surgery, 1980–2010. Iowa Orthop J 34, 197–203. 25328482 PMC4127720

[13] Niles SE, Balazs GC, Cawley C, Bosse M, Mackenzie E, Li Y, Andersen RC (2015) Translating research into practice: is evidence-based medicine being practiced in military-relevant orthopedic trauma? Mil Med 180(4), 445–453. 25826350 10.7205/MILMED-D-14-00296

[14] Elliott IS, Sonshine DB, Akhavan S, Slade Shantz A, Caldwell A, Slade Shantz J, Gosselin RA, Coughlin RR (2015) What factors influence the production of orthopaedic research in East Africa? A qualitative analysis of interviews. Clin Orthop Relat Res 473(6), 2120–2130. 25795030 10.1007/s11999-015-4254-5 PMC4419000

[15] Arksey H, O’Malley L (2005) Scoping studies: towards a methodological framework. International Journal of Social Research Methodology: Theory & Practice 8, 19–32.

[16] Daudt HM, van Mossel C, Scott SJ (2013) Enhancing the scoping study methodology: a large, inter-professional team’s experience with Arksey and O’Malley’s framework. BMC Med Res Methodol 13, 48. 23522333 10.1186/1471-2288-13-48 PMC3614526

[17] Levac D, Colquhoun H, O’Brien KK. 2010 Scoping studies: advancing the methodology. Implement Sci 5, 69. 20854677 10.1186/1748-5908-5-69 PMC2954944

[18] World Bank (2014) http://data.worldbank.org/about/country-and-lending-groups. Accessed June 30, 2014.

[19] Marx RG, Wilson SM, Swiontkowski MF (2015) Updating the assignment of levels of evidence. J Bone Joint Surg Am 97(1), 1–2. 25568387 10.2106/JBJS.N.01112

[20] Wright JG, Swiontkowski MF, Heckman JD (2003) Introducing levels of evidence to the journal. J Bone Joint Surg Am 85, 1–3. 12533564

[21] Cunningham BP, Harmsen S, Kweon C, Patterson J, Waldrop R, McLaren A, McLemore R (2013) Have levels of evidence improved the quality of orthopaedic research? Clin Orthop Relat Res 471(11), 3679–3686. 23846606 10.1007/s11999-013-3159-4 PMC3792258

[22] Murphy RF, Cibulas AM, Sawyer JR, Spence DD, Kelly DM (2015) Levels of evidence in the Journal of Pediatric Orthopaedics: update and comparison to the Journal of Bone and Joint Surgery. J Pediatr Orthop 35(7), 779–781. 25412071 10.1097/BPO.0000000000000362

[23] Yarascavitch BA, Chuback JE, Almenawer SA, Reddy K, Bhandari M (2012) Levels of evidence in the neurosurgical literature: more tribulations than trials. Neurosurgery 71(6), 1131–1137, discussion 1137–8. 22986592 10.1227/NEU.0b013e318271bc99

[24] Phillips J, Jergesen HE, Caldwell AM, Coughlin RR (2009) IGOT-The Institute for Global Orthopaedics and Traumatology: a model for collaboration and change. Tech Orthop 24(4), 308–311.

[25] Moher D, Pham B, Lawson ML, Klassen TP (2003) The inclusion of reports of randomised trials published in languages other than English in systematic reviews. Health Technol Assess 7(41), 1–90. 10.3310/hta7410 14670218

